# A pragmatic cluster randomised trial evaluating three implementation interventions

**DOI:** 10.1186/1748-5908-7-80

**Published:** 2012-08-30

**Authors:** Jo Rycroft-Malone, Kate Seers, Nicola Crichton, Jackie Chandler, Claire A Hawkes, Claire Allen, Ian Bullock, Leo Strunin

**Affiliations:** 1 Centre for Health Related Research, School of Healthcare Sciences, Bangor University, Ffriddoedd Road, Bangor, UK; 2 RCN Research Institute, School of Health and Social Studies, Warwick University, Coventry, UK; 3 Faculty of Health and Social Care, London South Bank University, London, UK; 4 Cochrane Collaboration, Summertown Pavilion, Oxford, UK; 5 National Clinical Guideline Centre (NCGC), Royal College of Physicians, St Andrews Place, London, UK; 6 former President of the Royal College of Anaesthetists, London, UK

## Abstract

**Background:**

Implementation research is concerned with bridging the gap between evidence and practice through the study of methods to promote the uptake of research into routine practice. Good quality evidence has been summarised into guideline recommendations to show that peri-operative fasting times could be considerably shorter than patients currently experience. The objective of this trial was to evaluate the effectiveness of three strategies for the implementation of recommendations about peri-operative fasting.

**Methods:**

A pragmatic cluster randomised trial underpinned by the PARIHS framework was conducted during 2006 to 2009 with a national sample of UK hospitals using time series with mixed methods process evaluation and cost analysis. Hospitals were randomised to one of three interventions: standard dissemination (SD) of a guideline package, SD plus a web-based resource championed by an opinion leader, and SD plus plan-do-study-act (PDSA). The primary outcome was duration of fluid fast prior to induction of anaesthesia. Secondary outcomes included duration of food fast, patients’ experiences, and stakeholders’ experiences of implementation, including influences. ANOVA was used to test differences over time and interventions.

**Results:**

Nineteen acute NHS hospitals participated. Across timepoints, 3,505 duration of fasting observations were recorded. No significant effect of the interventions was observed for either fluid or food fasting times. The effect size was 0.33 for the web-based intervention compared to SD alone for the change in fluid fasting and was 0.12 for PDSA compared to SD alone. The process evaluation showed different types of impact, including changes to practices, policies, and attitudes. A rich picture of the implementation challenges emerged, including inter-professional tensions and a lack of clarity for decision-making authority and responsibility.

**Conclusions:**

This was a large, complex study and one of the first national randomised controlled trials conducted within acute care in implementation research. The evidence base for fasting practice was accepted by those participating in this study and the messages from it simple; however, implementation and practical challenges influenced the interventions’ impact. A set of conditions for implementation emerges from the findings of this study, which are presented as theoretically transferable propositions that have international relevance.

**Trial registration:**

ISRCTN18046709 - Peri-operative Implementation Study Evaluation (POISE).

## Background

Implementation research is concerned with bridging the gap between evidence and practice through ‘the scientific study of methods to promote the systematic uptake of clinical research findings and other evidence-based practice into routine practice, and hence improve the quality…of healthcare’ [1]. Whilst the number of evidence-informed guidelines, frameworks, and standards are growing rapidly, their use in practice is frequently reported as being unpredictable, often slow, and complex [2-7]. This paper reports a large national implementation research trial to evaluate three strategies for the implementation of best practice recommendations for peri-operative fasting.

Several systematic reviews summarise the evidence about interventions for changing behaviour, using guidelines and research in practice, and quality improvement collaboratives [8-14]. Whilst a consistent message from these reviews is that the quality of implementation studies is generally poor, a number of strategies show some promise. Wallin [15] grouped guideline implementation strategies into the categories shown in Table 1.

**Table 1:** Effectiveness of interventions for guideline development

**Dissemination / educational strategies**	
Educational materials	Mixed effects
Conferences, courses	Mixed effects
Different education strategies	Mixed effects
Educational outreach visits	Effective
Mass media campaigns	Mostly effective
**Social interaction strategies**	
Interaction small-group meetings	Mostly effective
Feedback on performance	Mixed effects
Opinion leaders	Mixed effects
Multi-professional collaboration	Effective
**Decision support strategies**	
Reminders	Mostly effective
Computerised decision support	Mostly effective
**Organisational strategies**	
Introduction of computers into primary care to improve clinical performance	Mostly effective
Expanding professional roles	Mixed effects
Total quality management/quality improvement	Limited effects
Financial interventions	Effective
**Patient-orientated strategies**	
Patient mediated interventions	Mixed effects

Adapted from Wallin *et al*. 2009 and Grimshaw *et al*. 2004.

Findings from these systematic reviews show that interactive education approaches, audit and feedback, reminder systems, and opinion leadership may have some impact. Schouten *et al*. [13] also found that quality improvement collaborative approaches showed moderate positive results in some controlled studies. Additionally, it is suggested by Grimshaw *et al.*[10] that passive dissemination of guidelines should not be discounted because it offers a cheaper, feasible approach that may show effectiveness. They found that multi-faceted interventions were not necessarily more effective than single interventions [10]. Finally, the authors of all these reviews point out that very rarely are the cost consequences of implementation and quality improvement considered within study designs. A further critique of the evidence base is that there has been a lack of attention to implementation processes and theory within trials, which means that it is often difficult to determine why interventions may have worked (or not).

There is a growing and persuasive body of evidence that serves to highlight that the implementation of evidence into practice may be influenced by many factors, including those that could be considered organisational or contextual [16-20]. This highlights that implementation strategy development and the evaluation of implementation efforts need to capture contextual dimensions and pay attention to a broad range of influences.

### Fasting practice

Millions of people are admitted for elective surgery each year. In preparation for anaesthesia, fasting regimes are imposed in order to minimise the risk of regurgitation and aspiration of stomach contents [21]. Traditional fasting rules determine that patients are nil by mouth from midnight for a morning theatre list or have a light breakfast for an afternoon list. However, there is good quality robust research synthesised into national guidelines that recommend it is safe for healthy adult patients undergoing elective surgery to have water and clear fluids up to two hours before the induction of anaesthesia and food up to six hours prior to induction [21-24]. Despite a robust evidence base to guide practice, surveys show that prolonged fasting is common across the globe [25-27]. A UK survey of departments of anaesthesia revealed that 93 (62%) followed a fasting from midnight policy [28], whilst fluid intake until four, three, and two hours prior to surgery was reported as ‘acceptable’ under current guidelines at 40, 31, and 71 departments respectively. Prolonged fasting can lead to dehydration, electrolyte imbalance, nausea, and a reduced nutritional intake [29,30] and discomfort for patients [24].

No research was found reporting attempts to improve fasting times. This gap provided an opportunity to evaluate implementation interventions to promote the use of recommendations from a national guideline:

### Preoperative fasting in adults undergoing elective surgery – ‘the 2 and 6 rule’ [24]

· ‘2’ - Intake of water up to two hours before induction of anaesthesia.

· ‘6’ - A minimum preoperative fasting time of six hours for food (solids, milk, and milk-containing drinks).

· Postoperative resumption of oral fluids in healthy adults when fully awake.

### Summary

The evidence base for implementation provides some information about the strategies that might be more effective than others for implementing guidelines. However, it is not clear which strategies might be more or less effective in different contexts, with different clinical topics and professional groups. Therefore, our trial objective was to evaluate three interventions to implement fasting recommendations into practice with a focus on summative outcomes (duration of fasting) and the processes of implementation (intervention delivery, influences, and other types of impacts).

There have been calls for more robust implementation intervention studies [*e.g.,* 15], and to our knowledge this study is the first and largest implementation research trial to attempt to improve peri-operative fasting times.

## Methods

### Design

**Figure 1:**
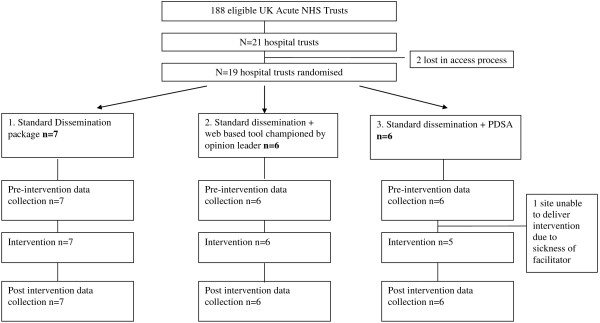
Flow chart from recruitment to post-intervention data collection.

This study was a pragmatic cluster randomised controlled trial (RCT) using time series with embedded mixed methods process and economic evaluation. The trial had three arms: standard dissemination (SD) of a guideline package; SD plus a web-based education package championed by an opinion leader, and 3) SD plus a Plan-Do-Study-Act (PDSA) approach. Hospital Trusts were randomised to one of the three implementation interventions. Data were collected eight months pre- and post-intervention. The intervention period was six months. The CONSORT flow diagram is shown in Figure 1.

### Theoretical framework

**Figure 2:**
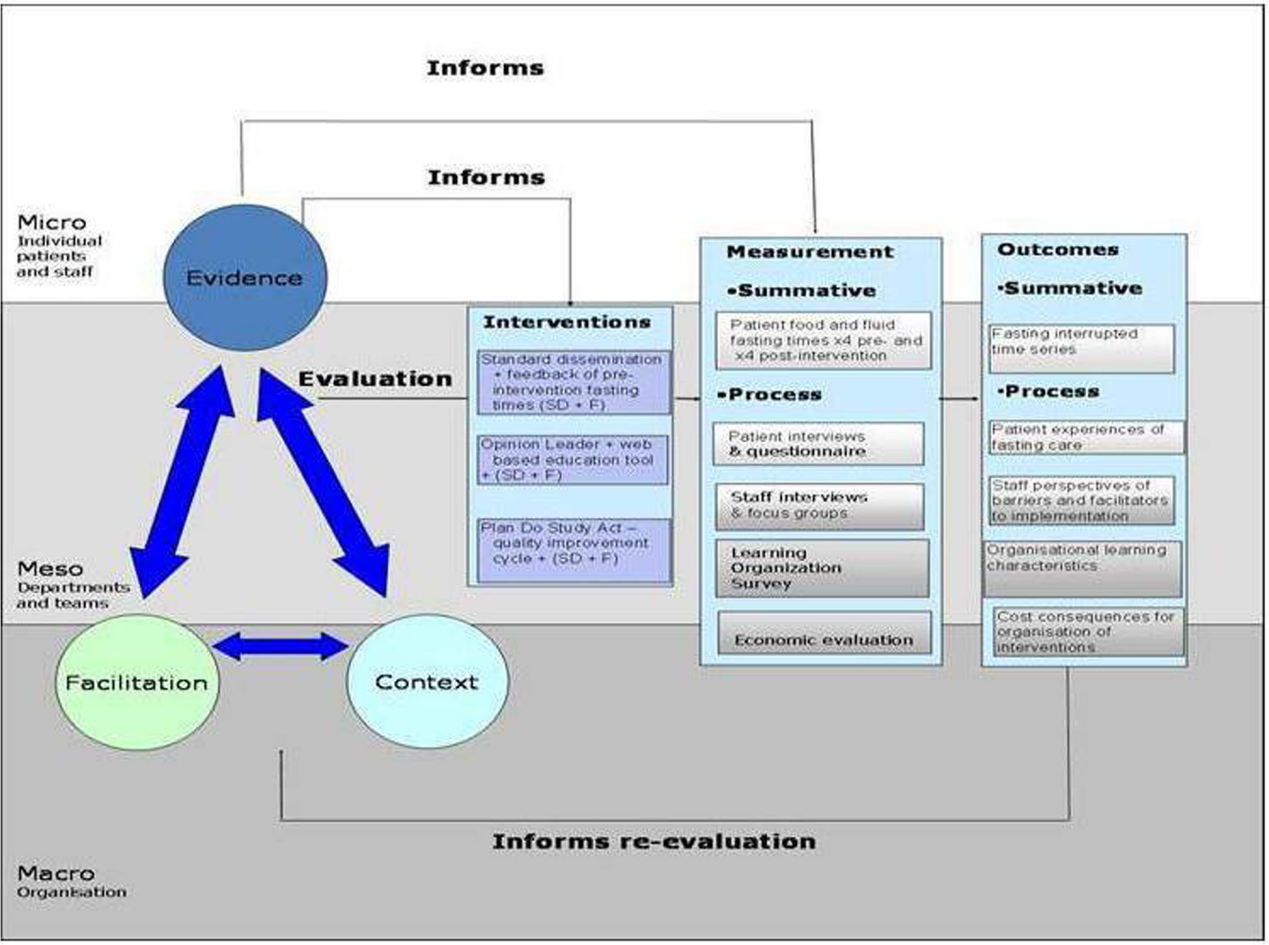
Theoretical framework.

The theoretical framework (Figure 2) developed for this study is based on the Promoting Action on Research Implementation in Health Services (PARIHS) framework [20,31]. Successful implementation (SI) is represented as a function (f) of the nature and type of evidence (E) (including research, clinical experience, patient experience, and local information), the qualities of the context (C) of implementation (including culture, leadership and evaluation), and the way the process is facilitated (F) (internal and/or external person who enables implementation processes); SI = f(E,C,F). The framework was used to incorporate interventions and to guide decisions about data collection, qualitative data analysis, and synthesis.

### Setting

All acute care NHS Trusts across the UK conducting elective surgery were invited to participate, but needed to be able to fulfil the following criteria:

1. there were a sufficient volume of suitable participants, *i.e*., at least 40 adult patients per timepoint undergoing a routine surgical operation as inpatient or day patient;

2. they provided gynaecological, orthopaedic, or general surgical services;

3. they would allow staff to participate in the project;

4. they would provide local investigators.

### Participants

Primary outcome data were collected from individual patients undergoing elective and routine general, orthopaedic, or gynaecological surgery.

### Inclusion criteria

1. Patients aged 18 and over.

2. Patients who could provide informed consent.

### Exclusion criteria

1. Patients who were critically ill.

2. Emergency or trauma patients because we were evaluating routine fasting regimes.

3. Patients who were unable to give informed consent

Duration of food fast was collected from all patients, but other secondary outcome data were collected from a sample of patients who participated in primary outcome data collection and a purposive sample of staff.

### Interventions

Trusts were randomised to one of the following interventions; two of which could be described as complex interventions. See Table 2 for a summary of intervention characteristics and expected mechanisms of action. The null hypothesis was that all interventions are equally effective.

**Table 2:** Intervention details

	**Intervention**		
	**Standard dissemination**	**Web-based resource**	**PDSA**
**Source**	Guideline	Guideline and RCN learning resource	Adapted from Modernisation Agency Improvement Leaders Guide
**Format**	Paper and CD	Computer	Package and facilitator led
**Target**	Trust	Multi-professional staff, individuals and/or groups	Multi-professional staff groups
**Delivered by**	Unknown	Local opinion leader	PDSA facilitator
**Duration**	Six months	Six months	Six months
**Number of events**	One	Multiple but not specified	Six meetings specified plus local audit activity
**Proximity to practice**	Remote	Arms length	Grassroots
**Setting**	Trust	Ward/theatre	Ward/theatre
**Intended mechanism of action**	Awareness raising	Social influence	Facilitation

#### Standard dissemination

SD of a guideline package, which included:

1. A copy of the RCN/RCA guideline, which included an overview of the guideline development process and those involved, recommendations, algorithm poster, and audit criteria.

2. A patient version of the guideline.

3. A PowerPoint presentation outlining some principles of guideline implementation.

We attempted to mirror, as far as possible, the dissemination process of the National Institute for Health and Clinical Excellence (NICE). The package was distributed once via the post-at the beginning of the intervention period and was targeted at the senior levels of the NHS Trust organisation. Packs were posted to named medical directors, nursing directors, clinical governance leads, and audit leads at each Trust. Packs were also sent to the English Strategic Health Authorities and the Health Boards in Northern Ireland, Wales and Scotland.

#### Standard dissemination of guideline package plus web-based educational package championed by an opinion leader(s)

A web-based resource was developed from the content of the guideline package, accessible to trusts allocated to this intervention. The web-based resource was interactive, incorporating educational tools such as self-check tests, working through clinical scenarios, and a patient digital story (http://www.rcn.org.uk/development/practice/perioperative_fasting).

The resource was championed by opinion leaders working in participating surgical areas. Opinion leaders were identified by key contacts at the NHS Trusts through a nomination process based on criteria developed from previous research [32-34]:

1. Does this person have credibility across different professional groups? *i.e.,* will different professional groups all take on knowledge from this person and respect their ability?

2. Do they have an authority and presence recognised by their colleagues?

3. Do they have good communication skills?

4. Do they treat all colleagues with respect?

5. Do they have the ability to convince colleagues about reducing fasting times through the intervention?

The selection of more than one opinion leader was permitted. Training on the use of the web-based resource was provided to opinion leaders at the start of the implementation phase.

#### 3. Standard dissemination of guideline package plus plan-do-study-act

The PDSA quality improvement approach includes making small changes and test cycles to see whether an improvement occurs in the system or process [35]. Critical to this intervention is the potential to collaborate, which in this study was possible at a local level between teams and individuals. This intervention also included a ‘diagnosis’ phase based on the Seven S Model [18,36,37]. A dedicated facilitator with relevant clinical and/or managerial experience was identified by each trust’s key contact. Facilitators had a one-day training session. The PDSA package used in this study is available on request. All trusts received their individual baseline mean food and fluid fasting times at the beginning of the intervention phase.

### Outcomes

#### Primary outcome

The primary outcome was the duration of fluid fast prior to induction of anaesthesia. Data on fasting were collected by the local investigators by asking patients pre-operatively when they last ate, when they last drank, and post-operatively when they had a first drink. This information was checked against reported information in their notes. These data were recorded on digitally read data sheets developed for this study (Additional File 1: Table S1) coded for the Trusts and for individual patients. Local investigators received training about how to collect these data. Data were collected four times pre-intervention and four times post-intervention, with up to two months interval between data collection points.

#### Secondary outcomes: Process evaluation

1. Patients’ experiences of fasting:

Questionnaire: A 17-item digitally read questionnaire (Additional File 2: Table S2) was developed and given to all patients who consented to duration of fasting data collection. Patients could return completed questionnaires via a sealed box on the ward or in a postage-paid envelope.

Interviews: Semi-structured audio-recorded interviews were conducted with patients from each Trust to explore their experiences of fasting, pre- and post-intervention. Ward staff identified potential patients for interview and the majority of patients were interviewed within three days post-operatively.

2. PDSA Facilitator and opinion leader experiences of implementation including activities, barriers and facilitators and perceived impact were collected through audio recorded semi-structured telephone interviews both pre- and post-intervention.

3. Key contact experiences of intervention implementation and impact including resource use were collected through audio-recorded semi-structured telephone interviews conducted pre- and three months post-intervention.

4. Staff experiences of fasting practice and any impacts of the study were collected through multi-professional audio recorded focus groups in a sample of the trusts that had been identified within each of the intervention arms as having made the largest change to fasting times, and those that had made the smallest/no/marginal change.

5. Organisational culture was evaluated through the administration of the Learning Organization Survey [38]. The questionnaire was distributed by local investigators to a convenience sample of doctors, nurses, health care assistants, operative department assistants and porters once pre-intervention period and once post-intervention.

6. Cost analysis of developing and implementing the three interventions from a national perspective (cost of rolling out a particular intervention across the NHS), and from the perspective of a single trust (cost of all activity and resource used by trust employees in implementation).

### Sample size

#### Power calculation

This was a complex, multi-level study design, including three intervention groups, fasting time being measured for each patient, with randomisation at Trust level.

Whilst fasting time was measured in each patient, comparing fasting times before and after the intervention was done at the Trust (randomised unit) level. Because patients will generally only be in hospital for surgery at one timepoint we could not measure the effect of the intervention on fasting time within individual patients. The number of patients in that Trust whose fasting time was measured will affect the precision of the fasting time estimated for an individual hospital.

The sample size calculation was based on information from an audit of fasting time [28]. The study had 80% power to detect an effect size of 2 (a difference of 4 hours and SD 2 hours) with a two-sided 5% significance level, which required six Trusts in each of the three intervention groups. These calculations were carried out using the sample size calculator for one way ANOVA in MINITAB13.

At the time of designing the study, there was very little information published about average fasting time, beyond reports of fasting times of around 10 hours (*e.g.,* Seymour [39] reported mean fasting times of 11.5 hours and 10.4 hours for elderly care patients), thus there was little on which to base for the sample size calculation. Given the apparent high reported mean and consistent claims of long fasting times, it appeared that we could be sure the mean fluid fasting time would substantially exceed the target of two hours prior to introduction of the guidelines. A national survey indicated traditional guidelines still prevailed [28]. If the additional effort of implementation beyond simply providing the guidelines is to be worthwhile, there needs to be a substantially greater reduction in fasting time and there was certainly scope for large impact. After the study had been designed, a report of fasting times for women having scheduled caesareans was published and reported a mean fluid fast of 11 hours (SD 3 hours) and a mean food fast of 13 hours (SD 2 hours) [40]. Breuer *et al*. [41] also found mean fluid fast times of 10 hours and mean food fast times of 15 hours. Given that current practice appeared to be so far away from the guideline recommendations, it seemed reasonable to expect the more intensive interventions would have a substantial impact on fasting time and would not otherwise be clinically worthwhile, hence a large effect size was used in the sample size calculation.

The project successfully recruited 19 Trusts. Trusts represented the four UK countries of England (15 Trusts), Scotland (2 Trusts), Wales (1 Trust) and Northern Ireland (1 Trust) and are referred to as Trust A, B, C *et al*. At each fasting time data collection point, a target of collecting fasting times from 40 patients was set.

### Randomisation

Each participating Trust was given an ID number. The randomisation schedule was computer-generated centrally and prepared by a statistician who was independent of the project team. Allocation was thus concealed and could not be foreseen in advance of, or during enrolment. Trusts were allocated to one of three implementation interventions, which resulted in six in two arms and seven in the other. Trusts were informed of their allocation by a project researcher as near as possible to the intervention period.

### Blinding

Blinding of local investigators, research fellows or trust staff to interventions was not possible because the intervention required their active participation. Patients were aware of the study, but not informed of the intervention allocated to the trust.

### Ethics

This study was approved by a multi-site ethics committee (06/MRE01/20). It took approximately 12 months before all of the trusts gave local research governance approval.

### Analysis

#### Numerical data

Digitally read duration of fasting data sheets and patient experience questionnaires were scanned, saved as comma separated variable files (csv files), and then imported into SPSS version 14 where data were cleaned before analysis. Analysis was conducted at the cluster level; for each Trust, mean fasting times were calculated at each assessment timepoint. At each timepoint, the differences in mean fasting times between the three intervention groups were compared using analysis of variance (ANOVA). A repeated measure ANOVA across the timepoint means for all trusts, within each intervention group, was conducted. The trend coefficient was not significantly different to zero: there was no evidence of trend over time pre-or post-intervention [42] therefore data were combined across timepoints (1 to 4 and 5 to 8) and simple pre- and post-interventions comparisons were conducted using t-tests. The significance level used for all tests was 5%. The effect size was calculated for each of the web-based and PDSA interventions compared to SD for change in fluid fasting time between pre- and post-intervention. Confidence intervals for the effect size are based on a non-central t distribution [43].

Patient experience questionnaires were analysed in SPSS using descriptive statistics, chi squared tests were used to compare characteristics pre- and post-intervention.

Learning organization survey data were entered into Excel and a random sample of 10% of the data entry was quality checked before being exported into SPSS version 14. Descriptive and inferential statistics were conducted.

### Qualitative data

Audio-recorded individual and focus group interviews were transcribed in full. Data were analysed within data set and managed in N*DIST 5 (pre-intervention) and NVIVO 7 (post-intervention). A combined inductive and deductive thematic analysis process was used whereby a sub-set of transcripts within a data set (*e.g.,* patient interviews pre-intervention) were analysed inductively by JC and CH and then an analysis framework developed with JRM, which was then applied to the remainder of the data in that set.

The theoretical framework guided the integration of findings across data sets.

## Results

### Participants and data collected

All 188 acute trusts in the four countries were invited to participate. Trust chief executives, medical and nursing directors (Binleys database) were sent a letter of invitation. A total of 19 Trusts were recruited and remained in the trial. We have no reason to believe that the characteristics of participating Trusts were different from any other NHS Trusts. However, given their willingness to participate, we have to assume they have an interest and therefore motivation to want to do something about their fasting times, which may have made them atypical of other non-participating trusts. Data were collected between November 2006 and February 2009 at a time where the NHS was undergoing major reform under a previous administration (see Table 3 for data collected). Anecdotal feedback from some sites that made further enquiries, but who then did not participate, would suggest that engaging in an additional initiative at a time of change was not feasible for them. Seven trusts were allocated to SD, six to SD plus web-based resource championed by an opinion leader, and six to SD plus PDSA. One PDSA trust did not implement the intervention due to staff sickness.

**Table 3:** Summary of data collected across timepoints and intervention groups

	**Pre-Intervention (2007 to 2008)**	**Post-Intervention (2008 to 2009)**	**Total**
Food fast duration information	1,435	1,777	3,212
Fluid fast duration information	1,440	1,761	3,201
Patient experience questionnaires	1,069	1,215	2,284
Local investigator audit		54	54
Interviews with key contacts	16	12	28
Interviews with facilitators and opinion leaders	9	12	21
Interviews with participants who were both key contacts and facilitators or opinion leaders	3		3
Interview with patients about their experiences	35	35	70
Completed Learning Organisation Surveys (LOS)	758	318	1,076
Focus Group participants (in five groups)		32	32

Not all trusts were able to collect data for all eight timepoints. Pre-intervention (because of lengthy governance procedures), three trusts had information from timepoint four only, four trusts had information from timepoints three and four, 12 trusts had information from more than two pre-intervention timepoints. Post-intervention, one trust had information from only one timepoint, one trust had information for two post-intervention timepoints, 17 trusts had information from more than two post-intervention timepoints.

### Duration of fluid and food fast pre- and post-intervention

#### Trends over timepoints

ANOVA showed no significant difference in mean over time for either food or fluid fast time at any Trust pre-intervention and post-intervention. Additionally within each intervention group there was no evidence of a trend across time pre-or post-intervention (Table 4).

**Table 4:** Intervention group across pre-and post-intervention timepoints

**Intervention**	**Pre-intervention**		**Post-intervention**	
	**Food ANOVA**	**Fluid ANOVA**	**Food ANOVA**	**Fluid ANOVA**
Standard dissemination	p = 0.981	p = 0.951	p = 0.872	p = 0.160
SD + web-resource/opinion leader	p = 0.410	p = 0.716	p = 0.536	p = 0.814
SD + PDSA	p = 0.958	p = 0.981	p = 0.748	p = 0.714

#### Pre-intervention fluid and food fasting times

Across all hospitals, information was gathered from 1,575 patients in total in the pre-intervention period (fluid fast time was missing for 135 patients and food fast time missing for 140 patients). There was no significant difference in the mean food fasting time across the four timepoints (ANOVA, p = 0.677), the overall mean food fasting time pre-intervention was 14.0 hours (95% CI 13.6, 14.4). Also, there was no significant difference in the mean fluid fasting time across the four timepoints (ANOVA, p = 0.877), the overall mean fluid fasting time pre-intervention was 9.63 hours (95% CI 8.67, 10.6). For 68.4% of patients the fluid fast exceeds six hours, and for 31.3% of patients it exceeds 12 hours in the pre-intervention period. The intracluster correlation for food fasting time pre-intervention was 0.027 and for fluid fasting was 0.12.

#### Post-intervention fluid and food fasting times

Across all Trusts information was gathered from 1930 patients in total in the post-intervention period (fluid fast was missing for 169 patients and food fast for 153 patients post-intervention). There was no significant difference in the mean food fasting time across the four timepoints (ANOVA, p = 0.951) or in the mean fluid fasting time across the four timepoints (ANOVA, p = 0.311). The mean food fast was 14.3 hours (95% CI 13.8, 14.8) and mean fluid fast 8.72 hours (95% CI 7.87, 9.57).

#### Comparing the three intervention groups pre-and post-intervention

In the pre-intervention period there was no significant difference in the mean food fast time between interventions (ANOVA, p = 0.662). All the intervention groups appeared to be similar with regard to food fast. There was also no significant difference in the mean fluid fast time in the pre-intervention period across the intervention groups (ANOVA, p = 0.506).

Post-intervention there was no significant difference in the mean food fast time between intervention groups (ANOVA, p = 0.641). In the post-intervention phase all the intervention groups appeared to be similar with regard to food fast. There was also no significant difference in the mean fluid fast time in the post-intervention period between the intervention groups (ANOVA, p = 0.751).

**Figure 3:**
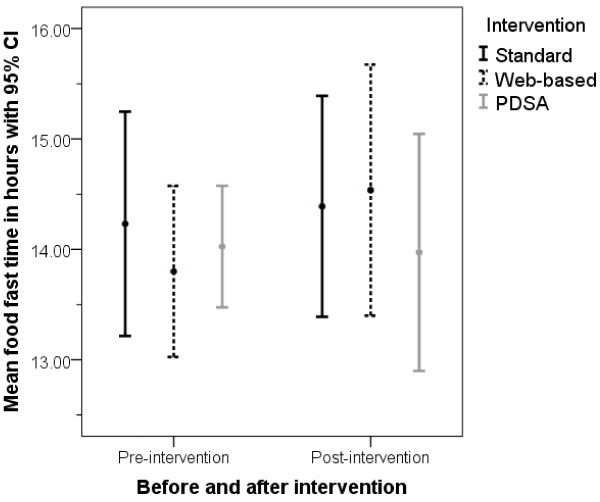
Mean and 95% CI for food fast time for each intervention group comparing pre- and post-intervention results.

**Figure 4:**
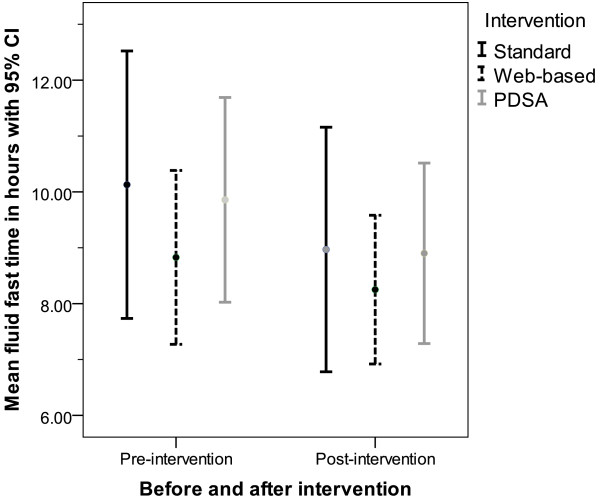
Mean and 95% CI for fluid fast time for each intervention group comparing pre- and post-intervention results.

The mean food and fluid fasting times for each intervention group at both pre- and post-intervention are shown in Table 5 together with the change in mean food and fluid fasting time (from pre-intervention to post-intervention) within each intervention group. The changes within each intervention group are small. For mean food fast, using a generalised linear model, neither the intervention nor the variable indicating pre/post-intervention is significant; Figure 3 compares the pre- and post-intervention food fast data for each intervention group. Similarly for mean fluid fast, using a generalised linear model, neither the intervention nor the variable indicating pre/post-intervention is significant; Figure 4 compares the pre- and post-intervention fluid fast data for each intervention group. Considering the change in fluid fasting time, the effect size for the web-based intervention compared to SD alone is 0.33 (95% CI −0.78, 1.42) and for PDSA compared to SD alone is 0.12 (95% CI −0.97, 1.21). These are small effect sizes; neither intervention shows the substantial impact beyond SD that would be required to reduce the mean fluid fasting time close to that recommended in the guidelines. For all three intervention groups, the post-intervention mean fluid fast time remains substantially above the guideline recommendation of two hours. Indeed, for 62.7% of patients the fluid fast exceeds six hours (the recommendation for food fast) and for 27.9% of patients it exceeds 12 hours.

**Table 5:** Mean food and fluid fasting times in hours with 95% confidence intervals for each intervention group pre- and post-intervention and for change in mean fasting time from pre-to post-intervention

**Intervention**	**Pre-intervention**		**Post-intervention**		**Change**	
	Food	Fluid	Food	Fluid	Food	Fluid
Standard dissemination	14.2	10.1	14.4	8.97	−0.16	1.16
	(95% CI 13.2, 15.2)	(95% CI 7.74, 12.5)	(95% CI 13.4, 15.4)	(95% CI 6.77, 11.2)	(95% CI −1.08, 0.76)	(95% CI −0.64, 2.96)
SD + web resource/opinion leader	13.8	8.83	14.5	8.25	−0.74	0.58
	(95% CI 13.0, 14.6)	(95% CI 7.27, 10.4)	(95% CI 13.4, 15.7)	(95% CI 6.92, 9.58)	(95% CI −1.99, 0.52)	(95% CI −1.06, 2.21)
SD + PDSA	14.0	9.86	14.0	8.90	0.05	0.96
	(95% CI 13.5, 14.6)	(95% CI 8.02, 11.7)	(95% CI 12.9, 15.0)	(95% CI 7.28, 10.5)	(95% CI −1.13, 1.24)	(95% CI −0.32, 2.23)
All intervention groups	14.0	9.60	14.2	8.91	−0.27	0.91
	(95% CI 13.7, 14.3)	(95% CI 9.00, 10.2)	(95% CI 13.9, 14.6)	(95% CI 8.46, 9.36)	(95% CI −0.80, 0.25)	(95% CI 0.16, 1.66)

#### Summary pre- and post-intervention fasting times

Both fluid and food duration of fasting data shows no significant change post-intervention for any of the three interventions. The effect sizes of the more intensive interventions compared to SD alone are small. Duration of fluid fast continues to substantially exceed the two-hour recommendation and food fast remains substantially longer than six hours regardless of the intervention used. Thus no particular intervention strategy was more effective than another.

### Cost analysis

A cost analysis of the three interventions was undertaken (see Additional File 3). Given that standard intervention was used in all three arms, the arm that received just standard intervention will by definition, have the lowest cost. One might assume this would be the most cost-effective intervention if the three approaches were shown to have equal effectiveness. Whilst the study did not detect a significant difference, it was not designed to demonstrate equivalence, and therefore it is not possible to say that SD is the most cost-effective approach. In fact, the variation in outcome within each intervention arm is likely to reflect the significant variation in activity, and therefore the resource use and cost, between the individual Trusts within each intervention arm.

### Patient experience

Findings from the questionnaires (n = 2,284, of which 1,069 were pre-intervention and 1,215 were post-intervention) and interviews (n = 70) relate to provision of information, management of delay and symptoms of fasting.

### Provision of information

Information about fasting was provided at least once for 89% of patients pre-intervention and for 91.6% of patients post-intervention. Information was sent by the hospital before admission for 55.8% of patients in the pre-intervention period and for 55.9% of patients post-intervention. One-fifth of patients (19.7% pre-intervention and 23.4% post-intervention) had information provided during the pre-admission clinic, but did not have information sent to them. For about a tenth of patients (10.7% pre-intervention and 10.6% post-intervention), they reported information about stopping eating and drinking only being provided on admission to the ward.

Patients liked clear consistent information, with repetitions being useful. Generally, patients were advised to fast as if they were going to be first on the list. Most patients reported ‘complying’ with instructions and in many cases were overly cautious by choosing to fast for longer than they needed to, 18.9% of patients pre-intervention and 18.1% post-intervention reported choosing to stop eating or drinking prior to their operation at different times to those recommended by the staff.

### Management of delay

Interview data showed that patients were tolerant of busy ward environments and often sympathetic to the need to prioritise the list order, sometimes rationalising that delays are due to emergencies or that organising the list is challenging. Patients reported being frustrated by not being reviewed if their operation was delayed.

### Symptoms of fasting

Approximately one-half of the patients completing the survey (46.5% pre-intervention and 44% post-intervention) felt hungry before their operation, however, the majority of patients (70.7% pre-intervention and 65.4% post-intervention) experienced thirst.

### The impact and process of implementation

Process evaluation data show a detailed picture of implementation processes within and across the 19 trusts and that the study impacted in other ways other than on the primary outcome. These findings are described below under the main elements of the conceptual framework: evidence, context, and facilitation.

### Impact

Table 6 summarises some of the impacts of the study had in trusts described by facilitators, opinion leaders and key contacts. The study may have had an impact on aspects of practice and service delivery that did not translate into changes to length of fast. Some of the influences on attempts to changing practice are outlined below (and will be described in detail in a separate publication).

**Table 6:** Summary of impact with examples

**Impact**	**Description**
Policy changes and development	Some trusts participating in the study had no Trust fasting policy. Some had fasting policies that were not consistent with guideline recommendations. For these trusts, the intervention period included the development of a policy (which in one Trust took six months to complete), and amending existing policy to ensure it was with the guideline recommendations.
Changes to information given to patients pre-operatively	Some trusts either developed or amended their patient information, including information provided in the letters that were sent to patients’ pre-operatively to make it clear what time individual patients could eat and drink up to (*i.e.,* a move away from traditional block fasting rules), and what exactly terms such as ‘clear fluids’ meant.
Introducing new approaches to communicating individual fasting times	Examples of different practical approaches to making patients and staff aware of the individual fasting times were reported. For example, the use of various tools to mark/record individual patient fasting times, such as paper cups, white boards and drug charts. Other practices included taking a more active approach to encouraging patients to drink up to two hours before anaesthesia.
Improved communication	Some staff reported that there had been improved communication between staff, and staff and patients about fasting times (although communication was also highlighted as a barrier to changing practice in some trusts).
Management of lists	In some trusts it was reported that there had been a review of operational list management to attempt to facilitate more individualised fasting times.
Raising awareness of fasting	It was reported that the project raised practitioners’ awareness of fasting practice in their units through informal and formal education sessions, meetings, web-based resources, data collection, role modelling.
Development of individuals	A number of staff reported personal and professional development as a result of taking on key contact and facilitator of PDSA roles.

### Evidence

The research underpinning the fasting recommendations could be judged as strong, coming from robust RCTs, and ‘badged’ by relevant UK Royal Colleges and Societies. Interview data suggests that most nursing and anaesthetists interviewed thought the evidence underpinning the recommendations was good and the guideline credible, for example:

"‘…I am quite happy about the evidence base … this guideline is not negotiable… you can’t say ‘well I don’t like this bit of it’…it’s based on best evidence.’ (Trust G, Key Contact and Change Agent [nurse])"

Issues were raised about the interpretation of some of the recommendations, such as what clear fluids meant, and the amount of water patients could drink.

Anaesthetists’ reaction to the evidence base tended towards conservative behaviour, which seemed to be based on prior experience where lists had worked well using traditional fasting rules, for example:

"‘… Why the extra aggro if there’s a sudden change in the list…I’ve been doing it x years and I’ve never had a cancellation because of that.. I personally… modify the rules. If somebody turns up having had a jug of water and it’s only one hour and 45 minutes I’ll go ahead and anaesthetise them. Other guys will say…let’s cancel, postpone.’ (Trust L, Anaesthetist Key Contact and Change Agent)"

### Context

Contextual factors were important influences on individual’s and trust’s capability to change practises in line with guideline recommendations, not least that it is challenging to change service delivery in a constantly changing environment. Three main findings are outlined here.

### Inter-professional issues

Inter-professional relationships were a significant emergent theme across all data. This incorporated different professional approaches, leadership, power and hierarchical structures, and professional cultures. Although we did not set out to specifically explore professional culture data, findings show that fasting practice was influenced by how the disciplines functioned together, sometimes bringing them into conflict because they had different objectives, ways of working and power bases.

"‘… The fasting guidelines are… embedded in so many different cultures that – so many different aspects of the organisation that they really are quite difficult to change.’ (Trust R Anaesthetist PDSA Facilitator)"

There was a professional struggle over fasting practice within Trusts. For example, one anaesthetist key contact (Trust J) sent out emails to medical staff promoting shorter fast times. Some of their colleagues replied ‘this is my theatre.’ Often, such behaviour resulted in nurses being caught in the middle of variances in practice between anaesthetists.

### Communication

Linked to inter-professional working, the nature and quality of communication between individuals, teams, and departments significantly impacted on fasting practice. In some trusts, communication between theatres and wards about delays was considered good. In others it was poor and seen as an area for improvement particularly with respect to revising and individualising fasting times.

"‘…We’ve had a phone call from theatre to say this patient’s been cancelled and you can feed and water them. Half an hour later we’ve had a phone call saying has she or he been fed and watered, she can go down so there’s been a big miscommunication or been told completely wrong....’ (Trust A Nurse, Focus group-SD)"

### Implementation context

The response rates to the Learning Organization Survey were low (18% pre-intervention and 7.4% post-intervention) and therefore findings interpreted with caution. General features to emerge included that the organisations in this study were perceived to be highly structured, rule-based organisations. Over 50% of responders did not feel innovative ideas were rewarded, and fewer than 60% of the responders believed they are required to upgrade and increase their knowledge. Less than 50% of responders did not feel an integral part of their Trust.

### Facilitation

Opinion leaders and PDSA facilitators within two intervention arms had the potential to take on facilitation roles (‘making things easier’). In reality, the enactment of these roles varied and linked to activities rather than the model of facilitation/change agency of the intervention. As such, fidelity to interventions was variable and as a research team did not intervene in the intervention phase. Skills and attributes and activities are summarised in Tables 7 and 8.

**Table 7:** Skills and attributes of opinion leaders and facilitators

**Attribute or skill**	**Description**
Authority	Through their position (role) and their seniority they had the status and autonomy to influence colleagues and decide how to do this. This attribute may have been particularly important in this study where fasting practice was not particularly viewed as a clinical priority.
Credibility	Often specified as clinical credibility, which in turn commanded respect of colleagues.
Drive, commitment, tenacity and enthusiasm	To see the project through and keep motivated and motivate others.
Change management and practice development skills, including:	These skills were seen as important for identifying facilitators and barriers, handling difficult situations, understanding ‘where people are coming from,’ and leadership in practice change. Both opinion leaders and facilitators reported working with teams.
· People management	
· Inter-professional working	
· Networking	
· Leadership	
· Education	
Communication skills	The ability to communicate well was perceived as contributing to the effectiveness of the skills and attributes described above.

**Table 8:** Implementation activities

**Activity**	**How operationalised**
Using existing structures or initiatives	For example, adding a discussion of fasting times to pre-list theatre meetings introduced as part of The Health Foundation Safer Patient Initiative or adding some information giving process (verbal or written) to pre-assessment clinic appointments.
Dissemination of information	Dissemination of the guideline to staff either on the intranet, via email or paper copies or the placement of the algorithm poster on staff information boards.
Sharing examples of good practice	Highlighting certain wards, departments and anaesthetists as role models.
Collection of local data	Some trusts collected data on fasting and/or patients’ views of fasting (separate from their involvement in the study).
Informal and formal education	Using real time practice opportunities such as anaesthetic rounds to educate staff, and more formally through education sessions and web tool use.
Identifying local leaders to work with/delegate to	Identifying and working through others within trusts to lead on practice change such as anaesthetic nurses, theatre co-ordinators, and surgical care practitioners.

### Summary

Whilst the research evidence underpinning fasting recommendations was strong and relatively uncontested, its translation into practice was challenging. Overall, there was no significant change to fluid and food fasting times pre- and post-intervention and no significant differences between the effectiveness of the three implementations. There were some significant decreases in fluid fast times within trusts, but in two trusts there was a significant increase in either fluid or fast times. These data present a complex, but realistic picture of implementation within acute care settings where multiple people, teams, and departments are involved.

## Discussion

This study was a large and complex evaluation of implementation interventions within acute care, the findings of which are relevant to the international community. Evidence use can impact in different ways. The gap between changing behaviour or practice, and how a change in practice or behaviour then translates into a change in (patient) outcomes is notoriously challenging to achieve. Within this study, whilst we did not observe significant positive changes to the primary outcome of duration of fluid fasting, other types of impact were achieved, which can be mapped along the continuum of research use that includes changes to awareness, knowledge and understanding, attitudes, perceptions, ideas, and practice and policy changes [44]. For example, trusts’ involvement in the project had raised awareness of the issue of fasting locally. Data also show that some participants believed that attitudes and ideas towards local fasting practice had shifted and that the project had ‘kick-started’ ideas. In some trusts, new policies and procedures were developed and there were changes to fasting practice. However, these impacts were not enough to result in changes to fasting outcomes over the time period studied. Methodologically, if we accept that evidence can have different types of impact, this raises questions about how these should/could be captured within implementation projects. There have been calls for ensuring that process evaluations are embedded within trials [10], and this study demonstrates why theory-led evaluation is important. Using theory facilitates a better understanding of impacts and outcomes, including and understanding of what, why, and how [45]

Process data show that the most influential mediators of practice change were inter-professional issues/tensions and communication, and a lack of clarity for the authority and responsibility for local fasting decisions (*e.g.,* when operating lists changed). These issues were evident irrespective of the allocation to intervention. Theoretically, the PDSA intervention had the potential to diagnose and ameliorate these issues, however within this study facilitator activities undertaken were more project related (*e.g.,* arranging education meetings) than process orientated (*e.g.,* teambuilding) [46,47]. Future change strategies in this area of practice would benefit from a focus on effective team working. More generally, there is still much to learn about what ‘good enough’ facilitation is, and what might work in different situations.

Theoretically, we had anticipated that more active interventions might have a greater impact than a passive strategy alone. Our findings suggest the effect size of the more active interventions compared to SD alone is small, thus supporting previous assertions that passive and simple interventions, may be as effective as active, and multi-faceted ones [10]. However, process data provide some more explanation. Closer analysis of the trust that made the greatest change to fasting times, for example, shows they were disappointed to have been allocated to SD, there was an existing commitment by the hospital to reduce fasting times, there was targeted and active dissemination of the guideline to relevant parts of the organisation, a number of key stakeholders took a leadership role in championing the issue, and the key contact led on multiple implementation related activities. These findings are supported by published studies showing that strategic buy-in, leadership, and active strategies may all be essential ingredients for successful implementation [31,48,49].

As researchers, our ability to ‘control’ intervention and implementation activities in this study was limited. Theoretically, the interventions were standardised in that all were ‘packaged’ and appropriate training provided. However, in reality, there was variability in the starting points of trusts (*e.g.,* some had no fasting policy), and therefore a difference in how different components of the interventions were implemented. For example, all PDSA intervention trusts had a start-up meeting, but not all continued to meet regularly throughout the intervention period as planned in the intervention package because of difficulties securing time away from practice. Therefore, within this study there are important questions about intervention fidelity and whether relevant staff and departments had insufficient exposure to the implementation interventions. However, there are questions to ask about if, and how, you could control activities in studies that are undertaken in the reality of complex and unpredictable clinical environments where researchers are working at arm’s length with local clinical/research communities. Hawe *et al*. [50] suggest we pay attention to the function and process of the intervention, rather than the standardisation of the intervention components themselves. Identifying the essential functions of an intervention, for example, facilitation, and examining the evidence for a fit with the theory of facilitation would provide an alternative perspective on standardisation and fidelity.

Within this study, some theoretical integrity was achieved by applying a theoretical framework that guided implementation intervention development, evaluation activities and for providing explanation. As outlined earlier, the underlying ‘theory’ of PARIHS is that SI = f(E,C,F). If we test the proposition with the findings of this study, the component that does not stand up to scrutiny is evidence. Fasting practice has a robust research evidence base, which was generally accepted by those participating in this study. Previous research has indicated that where there is strong research evidence, about which there is clinical consensus it is more likely to be used in practice [17,18]. In this study, the mediating effect of context (particularly the effect that teams and professional groups had on operating list management) was stronger, and facilitative activities used in this study did not overcome these.

### Limitations

The interrupted time series did not have all data at all timepoints for all trusts, mainly due to the delays in getting research governance approval from participating Trusts. However, because there were no significant time trends pre-or post-intervention, and thus all four pre-and all four post-timepoints were combined, the impact of this missing data was minimised.

The primary outcome was duration of fluid fast prior to induction of anaesthesia. This together with food fast time was collected by the local investigator asking patients when they last drank and ate, supported by data in patients’ notes. It is possible the times recorded were not always recalled or recorded completely accurately.

The intervention period was six months, which limited the amount of activity that was possible before post-intervention outcome data was collected. The four post-intervention timepoints did not show any trends over time, suggesting that a longer intervention period may not have led to improved fasting times. However, evidence is lacking about how long an intervention period is needed to make changes to practice and service delivery, particularly ones that sustain.

The size of this study—19 Trusts split over three intervention groups—may be seen as a limitation. The study was designed to be adequately powered to detect an effect size that was large because the evidence available at the time of designing the trial suggests fluid fasting times are far too long and would need to reduce substantially more for the intensive intervention than by SD if the effort required was to have an impact on fluid fasting time that was beneficial to patients.

As a cluster trial, to have a substantial impact on the power of the study would require additional Trusts, not simply more observations within the existing Trusts. However, to recruit and maintain Trusts in a study of this type is complex, costly, and very time-onsuming. The evidence from surveys suggests that fluid fasting times have not substantially decreased since the guidelines were published and disseminated in 2004 [39,40]; thus, our assumption that fluid fasting time needs to substantially reduce is still supported by other studies. Whilst it is important in designing studies to be realistic about the effect size that can be achieved, we also need to strive to detect effects of clinical worth not simply those that are statistically significant. The more intensive interventions considered in this study would need to show a substantial reduction in fluid fasting time beyond that of SD to be of noticeable benefit to patients.

Intervention fidelity was difficult to ensure and evaluate. We were aware that different teams implemented their intervention in different ways, and we attempted to capture this through the process evaluation. For example, some PDSA groups appeared to follow the process closely, others were less engaged and followed it in a more *ad hoc* manner. Thus, the intervention ‘dose’ is likely to be different between the groups. As a pragmatic RCT, tailoring of interventions is a recognised component [51] and viewed as making the intervention more feasible to implement in the real world.

## Conclusions

This study is one of the first national RCTs with an embedded process evaluation conducted within acute care in the field of implementation research. Evaluating implementation in the context of fasting practice provided a useful vehicle with which to expose some of the general issues and challenges faced when implementing evidence into practice. The issues uncovered in this study have important international implications because they have helped to further articulate the complex processes embedded in implementation as well as its evaluation.

There are a set of conditions and antecedents for implementation that emerge from the findings of this study, which we have developed into a number of propositions:

1. Implementation is more likely to be successful in cases where the topic/issue is a strategic and organisational priority. If the issue is not a priority, the active engagement of individuals and departments is mediated by for example, a lack of dedicated human and financial resource, and resultant implementation will be patchy.

2. A historical lack of clear leadership, structure, and process for local guideline dissemination and implementation, in which staff are unclear about their responsibilities, will negatively impact on an organisation’s ability to routinely use guideline recommendations.

3. Robust and believable evidence is not always sufficient to change decision making and practice, therefore implementation interventions and efforts need to extend beyond individual decision making (at least for certain clinical topics) and take account of the systemic interconnections between individuals, teams and organisations.

4. In areas where there is more effective teamwork with clear communication, practice change will be easier to achieve.

5. New improvement and implementation projects have a higher chance of success if they are embedded into existing programmes and structures.

6. Change agent effectiveness is a function of the protected space and dedicated time to fulfil the role, *i.e.,* it has to be part of the ‘day job.’

7. Change agents will be more effective if they have people management skills, work collaboratively, handle difficult situations and people with diplomacy, understand where people ‘are coming from,’ develop and motivate a team, including effective and considerate delegation of work, using team members skills well. This is a skilled role at which some excel, and some find more challenging.

8. Evaluations of implementation interventions that capture different types of impacts over the course of the study/programme are more likely to provide a realistic picture of knowledge use, and intended and unintended consequences. Clearly, how one proceeds to evaluate these impacts will be dependent on the definition of knowledge and use.

9. Complex interventions such as implementation interventions need to be deconstructed to gain a greater understanding of the linkages between the active components/mechanisms of action and the impact on both process and summative outcomes.

It is likely that these propositions will be theoretically transferable to other implementation studies, particularly when considered alongside the growing empirical and theoretical evidence base about the successful ingredients for successful implementation.

## Competing interests

The authors declare they have no competing interests.

## Authors' contributions

JRM conceived the study, and led the design of the study with KS, IB, and NC. JRM, KS, and IB secured funding. JRM supervised all aspects of the study, with input from KS. IB supervised the cost consequence analysis. NC led and conducted the quantitative data analysis. JC and CH coordinated and took the lead role in data collection and analysis, and commented on drafts of the paper. CA provided a patient perspective throughout the conduct of the study including the development of patient related materials and data collection processes. LS provided a clinical perspective on the project as an anaesthetist. JRM drafted the paper, KS, NC, and CH wrote sections of the paper and KS, CH, JC, CA, IB commented and approved the final manuscript.

## Supplementary Material

Additional file 1 **Table S1.** Data recording sheet for fasting time. Click here for file

Additional file 2 **Table S2.** Digitally read patient experience questionnaire. Click here for file

Additional file 3 **Table S3.** Cost analysis. Click here for file
