# Supplementary material for: A pragmatic cluster randomised trial evaluating three implementation interventions
Source: Implement Sci. 2012 Aug 30;7:80. doi: 10.1186/1748-5908-7-80 (PMC3457838; doi:10.1186/1748-5908-7-80)
Supplement: Additional file 1 — Table S1. Data recording sheet for fasting time. [file 1748-5908-7-80-S1.pdf]

PoISE - Fasting Data Collection Sheet

Peri-operative (fasting guideline) implementation study evaluation

DO NOT WRITE IN THIS SHADED AREA

INSTRUCTIONS

- This form will be read by a machine
- Use an HB pencil or black pen
- Mark like this

Please complete the following information:

Date of operation (DD/MM/YY) ___ ___ / ___ ___ / ___ ___

PATIENT ID NO. ☐☐☐☐ **SITE NO.** ☐☐

INVESTIGATOR/RESEARCHER NO. ☐☐ 1 2 3 4 5 6 7 8 9 10

WARD/DAY UNIT NO. ☐☐ 1 2 3 4 5 6 7 8 9 10

1. **Was the operation cancelled?** (Cancelled here means operation did not happen on the day it was planned) Yes ▭ Do not complete the rest of this form. No ▭ Go to question 2.

2. Mark the closest approximate days and times.

FOOD FAST COMMENCED (NO SOLIDS)		COMPLETE FAST COMMENCED (NBM)		INDUCTION OF ANAESTHETIC		FIRST DRINK POST OPERATIVE (i.e. MORE THAN SIPS)	
Sun Mon Tue Wed Thu Fri Sat		Sun Mon Tue Wed Thu Fri Sat		Sun Mon Tue Wed Thu Fri Sat		Sun Mon Tue Wed Thu Fri Sat	
00:00	12:00	00:00	12:00	00:00	12:00	00:00	12:00
:15	:15	:15	:15	:15	:15	:15	:15
:30	:30	:30	:30	:30	:30	:30	:30
:45	:45	:45	:45	:45	:45	:45	:45
1:00	13:00	1:00	13:00	1:00	13:00	1:00	13:00
:15	:15	:15	:15	:15	:15	:15	:15
:30	:30	:30	:30	:30	:30	:30	:30
:45	:45	:45	:45	:45	:45	:45	:45
2:00	14:00	2:00	14:00	2:00	14:00	2:00	14:00
:15	:15	:15	:15	:15	:15	:15	:15
:30	:30	:30	:30	:30	:30	:30	:30
:45	:45	:45	:45	:45	:45	:45	:45
3:00	15:00	3:00	15:00	3:00	15:00	3:00	15:00
:15	:15	:15	:15	:15	:15	:15	:15
:30	:30	:30	:30	:30	:30	:30	:30
:45	:45	:45	:45	:45	:45	:45	:45
4:00	16:00	4:00	16:00	4:00	16:00	4:00	16:00
:15	:15	:15	:15	:15	:15	:15	:15
:30	:30	:30	:30	:30	:30	:30	:30
:45	:45	:45	:45	:45	:45	:45	:45
5:00	17:00	5:00	17:00	5:00	17:00	5:00	17:00
:15	:15	:15	:15	:15	:15	:15	:15
:30	:30	:30	:30	:30	:30	:30	:30
:45	:45	:45	:45	:45	:45	:45	:45
6:00	18:00	6:00	18:00	6:00	18:00	6:00	18:00
:15	:15	:15	:15	:15	:15	:15	:15
:30	:30	:30	:30	:30	:30	:30	:30
:45	:45	:45	:45	:45	:45	:45	:45
7:00	19:00	7:00	19:00	7:00	19:00	7:00	19:00
:15	:15	:15	:15	:15	:15	:15	:15
:30	:30	:30	:30	:30	:30	:30	:30
:45	:45	:45	:45	:45	:45	:45	:45
8:00	20:00	8:00	20:00	8:00	20:00	8:00	20:00
:15	:15	:15	:15	:15	:15	:15	:15
:30	:30	:30	:30	:30	:30	:30	:30
:45	:45	:45	:45	:45	:45	:45	:45
9:00	21:00	9:00	21:00	9:00	21:00	9:00	21:00
:15	:15	:15	:15	:15	:15	:15	:15
:30	:30	:30	:30	:30	:30	:30	:30
:45	:45	:45	:45	:45	:45	:45	:45
10:00	22:00	10:00	22:00	10:00	22:00	10:00	22:00
:15	:15	:15	:15	:15	:15	:15	:15
:30	:30	:30	:30	:30	:30	:30	:30
:45	:45	:45	:45	:45	:45	:45	:45
11:00	23:00	11:00	23:00	11:00	23:00	11:00	23:00
:15	:15	:15	:15	:15	:15	:15	:15
:30	:30	:30	:30	:30	:30	:30	:30
:45	:45	:45	:45	:45	:45	:45	:45

S2121/F071106

continue overleaf

© Speedwell Computing Services, Tel: +44 (0) 1933 445820 enquiries@speedwell.co.uk www.speedwell.co.uk

Please mark the box that corresponds to the correct answer.

3. Was the operation delayed from planned time?
   - Yes ☐ Go to Q4
   - No ☐ Go to Q7

4. If the operation was delayed for more than two hours, did the patient have a drink of clear fluids while they were waiting?
   - Yes ☐ Go to Q5 and follow the instructions
   - No ☐ Go to Q6
   - Don't know ☐ Go to Q6

5. Please fill out a second data collection sheet with the revised start of complete fast (NBM) time and complete that form. You do not need to fill out the rest of this form. Please return both forms for this patient to the PoISE team researchers.

6. If the operation was delayed for more than two hours, was an intravenous infusion set up for hydration while the patient was waiting?
   - ☐ Yes
   - ☐ No
   - ☐ Don't know

7. What sort of case was this patient?
   - ☐ Elective Day Surgery
   - ☐ Elective Inpatient

8. Which type of surgical procedure was performed?
   - ☐ Orthopaedic
   - ☐ Gynaecological
   - ☐ General

9. Was the post operative drink delayed?
   - No ☐ Go to Q11
   - Don't know ☐ Go to Q11
   - Yes ☐ Go to Q10

10. Why was the post operative drink delayed?
    - ☐ Patient refused
    - ☐ Patient was vomiting
    - ☐ On medical instruction
    - ☐ Reason not clear

11. Did the patient aspirate during the peri-operative period (please check anaesthetic notes)?
    - Yes ☐
    - No ☐
    - Information not available ☐

Please mark the time measurement period for which this data has been collected

T1 ☐ T2 ☐ T3 ☐ T4 ☐ T5 ☐ T6 ☐ T7 ☐ T8 ☐

If there is any missing data on this sheet please mark all that apply

- ☐ There is missing data from the patient's case notes
- ☐ I have been unable to locate the patient's case notes to complete data collection
- ☐ I was unable to have access to the patient after they were consented
- ☐ I was not available to collect all of the data for this consented patient

S2121/R071106

© Speedwell Computing Services, Tel: +44 (0) 1933 445820 enquiries@speedwell.co.uk www.speedwell.co.uk
