# Supplementary material for: A pragmatic cluster randomised trial evaluating three implementation interventions
Source: Implement Sci. 2012 Aug 30;7:80. doi: 10.1186/1748-5908-7-80 (PMC3457838; doi:10.1186/1748-5908-7-80)
Supplement: Additional file 2 — Table S2. Digitally read patient experience questionnaire. [file 1748-5908-7-80-S2.pdf]

DO NOT WRITE IN THIS SHADED AREA

Following to be completed by the local investigator -

- Patient ID No.
- Site No.
- Ward/Day Unit No.
- Time Point

PATIENT ID NO.				WARD/DAY UNIT NO.		SITE NO.	
0	0	0	0	0	0	0	0
1	1	1	1	1	1	1	1
2	2	2	2	2	2	2	2
3	3	3	3	3	3	3	3
4	4	4	4	4	4	4	4
5	5	5	5	5	5	5	5
6	6	6	6	6	6	6	6
7	7	7	7	7	7	7	7
8	8	8	8	8	8	8	8
9	9	9	9	9	9	9	9

TIME POINT

T1 ☐ T2 ☐ T3 ☐ T4 ☐ T5 ☐ T6 ☐ T7 ☐ T8 ☐

PoISE - PATIENT QUESTIONNAIRE

INSTRUCTIONS

- This form will be read by a machine
- Use an HB pencil or black pen
- Mark like this ■

1. When were you given information about when to **stop eating and drinking** (fast) for your operation? (Please mark **all** that apply).

☐ The hospital sent me some information before I came into hospital (e.g. with the letter giving you a date for your operation).

☐ At the pre operative assessment clinic*.

☐ Once I was admitted to the hospital ward for my operation.

☐ Other please specify:

**A pre operative assessment clinic is an outpatient clinic where you are assessed for your operation. This is not usually the clinic when the specialist sees you to decide what is wrong and what treatment you need.*

Please rate how much you agree or disagree with the following statements. Please mark ONE option for each statement.

	Strongly agree	Agree	Neither agree nor disagree	Disagree	Strongly disagree	Don't remember
2. Different healthcare staff gave me different information about when I should stop **eating** before my operation.	☐	☐	☐	☐	☐	☐
3. I felt clear about when I should stop **eating** before my operation.	☐	☐	☐	☐	☐	☐
4. I felt hungry while I was not **eating** before my operation.	☐	☐	☐	☐	☐	☐
5. I felt thirsty while I was not **drinking** before my operation.	☐	☐	☐	☐	☐	☐
6. Different healthcare staff gave me different information about when I should stop **drinking** before my operation.	☐	☐	☐	☐	☐	☐
7. I felt confused about when I should stop **drinking** before my operation.	☐	☐	☐	☐	☐	☐
8. I felt thirsty after my operation.	☐	☐	☐	☐	☐	☐
9. I was given a drink after my operation as soon as I felt I wanted one.	☐	☐	☐	☐	☐	☐

S2284/F071106

© Speedwell Computing Services +44 (0)1933 445820 enquiries@speedwell.co.uk www.speedwell.co.uk

Please turn over

DO NOT WRITE IN THIS SHADED AREA

If you are currently taking any daily medication please answer question 10. If not, please tick the N/A box and go to question 11.

	Yes	No	Don't Know	N/A
10. I had all my medication as usual while I was not eating and drinking before my operation.	☐	☐	☐	☐

11. While you were not eating and drinking before your operation did you do any of the following? (Please mark any that apply).

- ☐ Sucked an ice cube
- ☐ Chewed gum
- ☐ Cleaned my teeth
- ☐ Had sips of water
- ☐ Ate a sweet/sweets
- ☐ Used lip moisturiser (e.g. lipsil)
- ☐ Used mouthwash
- ☐ Ate something else
- ☐ Had a drink

12. Did you choose to stop eating or drinking before your operation at different times from those recommended by the staff looking after you?

- ☐ Yes — If yes, please tell us the reason for your decision
- ☐ No

If your operation was delayed* please answer questions 13 and 14.
If not, please go to question 15.
**Delayed here means you still had your operation on the planned day but it was later than you were originally told you would go to theatre.*

	Strongly agree	Agree	Neither agree nor disagree	Disagree	Strongly disagree
13. I was happy about the way staff kept me up to date about what was happening when my operation was delayed.	☐	☐	☐	☐	☐
14. I was not given clear information about whether I could **drink** whilst I was waiting for my operation.	☐	☐	☐	☐	☐

15. Do you have any medical condition that can be affected by not eating or drinking (e.g. diabetes).

- ☐ Yes, Please answer Q16 &17
- ☐ No, You don't need to answer any more questions. Thank you for completing this questionnaire.

If you answered yes to question 15 above, please answer overall how you felt your condition(s) was/were treated.

	Strongly agree	Agree	Neither agree nor disagree	Disagree	Strongly disagree
16. Staff discussed treatment decisions about my condition(s) with me.	☐	☐	☐	☐	☐

17. If you would like to tell us what condition or conditions you have that are affected by not eating and drinking, please write them here.

S2284/R071106

Thank you for taking part in this research study and completing this questionnaire. Please put it in the envelope and put it in the collection box on the ward.
