# Supplementary material for: A pragmatic cluster randomised trial evaluating three implementation interventions
Source: Implement Sci. 2012 Aug 30;7:80. doi: 10.1186/1748-5908-7-80 (PMC3457838; doi:10.1186/1748-5908-7-80)
Supplement: Additional file 3 — Table S3. Cost analysis. [file 1748-5908-7-80-S3.doc]

Additional File 3

Cost analysis

We have estimated the mean cost for each intervention arm and used the information we have on the variability in activities to estimate the uncertainty around that cost estimate.

*Standard dissemination:* The national cost of standard dissemination (short guideline, a poster, patient information leaflet, and implementation PowerPoint guide) assuming the costs of guideline development are already met, but that designing, editing, reproducing, and posting need to be met was £6,531 when applied to 170 acute trusts. The total cost per pack is £7.66 and therefore the cost per Trust would be £38.42.

*Web-resource/opinion leader*: The cost of providing 170 acute trusts with implementation support through a web-based resource championed through opinion leadership would be £67,300. This includes development costs for the tool (which for this project were in-house costs, in other cases external agencies may have to be used which are likely to be three times higher), publicity materials, training materials and opinion leader time and activity. The total fixed cost was £11,405 giving a mean cost of £1,828 over the six Trusts within the study but a mean cost of £65 per Trust if the intervention were to be used nationally by all 170 acute Trusts.

*PDSA*: At a national level the cost of providing 170 acute trusts with implementation support through PDSA would be £153,700. The main cost driver is the need for regular meetings that are attended by up to 10 people. Additionally, a large proportion of this cost was external support for a first PDSA meeting; a different type of PDSA model may not incur these extra costs. The cost per trust may vary between £3,115 for a DGH with 8 wards, using a Senior Nurse Team Manager to facilitate the PDSA group and holding an average of 3 meetings over 6 months with 5 attendees, to £17,754 for a large teaching hospital with 12 surgical wards having monthly meetings facilitated by a Consultant who undertakes all the activities prescribed within the PDSA model. The ongoing costs would be £483 and £2,556 per month respectively.
